# Effectiveness of Mindfulness-Based Stress Reduction on Mental Health and Psychological Quality of Life among University Students: A GRADE-Assessed Systematic Review

**DOI:** 10.1155/2024/8872685

**Published:** 2024-02-20

**Authors:** Yuanqing Pan, Fusen Li, Haiqian Liang, Xiping Shen, Zhitong Bing, Liang Cheng, Yi Dong

**Affiliations:** ^1^Tianjin Vocational and Technical Normal University, Campbell China Network, Dagu Nan Lu, Hexi, Tianjin 300222, China; ^2^Department of Neurosurgery, Characteristic Medical Center of Chinese People's Armed Police Force, Tianjin, China; ^3^Institute of Epidemiology and Health Statistics, School of Public Health, Lanzhou University, 199 Donggang West Road, Lanzhou 730000, Gansu, China; ^4^Institute of Modern Physics, Chinese Academy of Sciences, 509 Nanchang Road, Chengguan 730000, Lanzhou, China; ^5^School of Computer Science, Beijing University of Posts and Telecommunications, Xitucheng Road, Haidian 100876, Beijing, China; ^6^Tianjin Medical College, School of Pharmacy and Biotechnology, Department of Traditional Chinese Medicine, Liulin Road, Hexi, Tianjin 300222, China

## Abstract

**Background:**

Psychological distress is a progressive health problem that has been linked to decreased quality of life among university students. This meta-analysis reviews existing randomized controlled trials (RCTs) that have examined the effects of mindfulness-based stress reduction (MBSR) on the relief of psychosomatic stress-related outcomes and quality of life among university students.

**Methods:**

The PubMed, EMBASE, Web of Science, PsycINFO (formerly PsychLit), Ovid MEDLINE, ERIC, Scopus, Google Scholar, ProQuest, and Cochrane Library databases were searched in November 2023 to identify the RCTs for analysis. Data on pathology (anxiety, depression, and perceived stress), physical capacity (sleep quality and physical health), and well-being (mindfulness, self-kindness, social function, and subjective well-being) were analyzed.

**Results:**

Of the 276 articles retrieved, 29 met the inclusion criteria. Compared with control therapies, the pooled results suggested that MBSR had significant effects, reducing anxiety (SMD = −0.29; 95% CI: −0.49 to −0.09), depression (SMD = −0.32; 95% CI: −0.62 to −0.02), and perceived stress (SMD = −0.41; 95% CI: −0.60 to −0.29) and improving mindfulness (SMD = 0.34; 95% CI: 0.08 to 0.59), self-kindness (SMD = 0.57; 95% CI: 0.30 to 1.12), and physical health (SMD = −0.59; 95% CI: −1.14 to −0.04). No significant differences were observed in sleep quality (SMD = −0.20; 95% CI: −0.06 to 0.20), social function (SMD = −0.71; 95% CI: −2.40 to 0.97), or subjective well-being (SMD = 0.07; 95% CI: −0.18 to 0.32). The quality of the evidence regarding sleep quality and physical health outcomes was low.

**Conclusions:**

MBSR therapy appears to be potentially useful in relieving functional emotional disorders. However, additional evidence-based large-sample trials are required to definitively determine the forms of mindfulness-based therapy that may be effective in this context and ensure that the benefits obtained are ongoing. Future studies should investigate more personalized approaches involving interventions that are tailored to various barriers and students' clinical characteristics. To optimize the effects of such interventions, they should be developed and evaluated using various designs such as the multiphase optimization strategy, which allows for the identification and tailoring of the most valuable intervention components.

## 1. Introduction

Mindfulness therapy is a standardized psychological intervention that aims to reduce stress, encourage mindful thinking habits, and allow recipients to manage difficult emotional processing. It focuses on the concentration of one's attention in the moment, nonsubjective judgment, and openness to accepting personal experience and involves corresponding behavioral training, such as attention training, body scanning, and sitting meditation [1]. Previous research on psychological interventions has found that mindfulness therapy can help reduce stress among university students and affects their overall quality of life and the psychological functions to which they adapt [2].

The theoretical support and the understanding of the operating mode are reasonable but still have differences. Thus, the mental health benefits, potential effects, and limitations of mindfulness therapy for university students warrant further examination. Although education and social support can be effective in preventing and treating the underlying psychological problems among university students with poor mental health, such interventions do not always affect or improve psychological well-being [3, 4].

The most prevalent psychological symptoms among university students are anxiety, mental stress, and emotional distress, which can result in decreased functioning in the context of academic and interpersonal stress. Global mental health surveys conducted by the World Health Organization (WHO) indicate that mental disorders are highly prevalent among university students, with 12-month prevalence rates ranging from 20.3% to 45% of university students over the age of 18 [5–7]. The prevalence rates of insomnia, depressive symptoms, and anxiety symptoms were 37.80%, 48.20%, and 36.70%, respectively, among Chinese university students during the COVID-19 pandemic [8]. In a randomized controlled trial with 11,169 university students conducted by the WHO, students had increasing intentions to use mental health services: The results indicated lifetime use rates of 12.6% and 7.3% for psychotherapy and medication for emotional problems, respectively. Suicidal thoughts and behaviors and nonsuicidal self-injury within the preceding 12 months were also common, with 21.1% of students in the sample reporting suicidal ideation, 10.6% reporting suicide plans, 0.4% reporting suicide attempts, and 6.8% reporting nonsuicidal self-injury [5]. Furthermore, one previous study found that high-compliance mental health services had a significant effect on students' intention to seek mental health services in the next semester [9–11].

Evidence indicates that mindfulness therapy can result in a significant reduction in burnout, distress, anxiety, depression, and stress and a significant improvement in life satisfaction, positive affect, gratitude, self-compassion, and mindfulness among university students [12, 13]. However, although some studies show that mindfulness-based stress reduction (MBSR) can help relieve psychological symptoms and improve quality of life among university students, there is no reliable evidence that this type of intervention can help prevent or treat psychological abnormalities and, thereby, improve university students' subjective well-being. In addition, there are disparities between consensus-based conclusions regarding MBSR and clinical recommendations.

Some studies have used evidence-based medicine approaches to examine the effects of MBSR treatment on university students' psychological conditions. A recent study revealed that an MBSR intervention group showed statistically significantly fewer symptoms of stress and distress and had higher mindfulness than the control group. Previous studies of MBSR have emphasized its potential benefits for university students, such as minimizing perceived stress, preserving emotional stability, enhancing social functioning, and increasing the self-attractiveness of the group [14–17]. This study adopted the Cochrane systematic review method to assess the effect of psychological interventions and the overall impact of MBSR treatment on the psychological condition of university students, aiming to provide a scientific basis for the clinical practice of MBSR treatment. Our systematic review aimed to assess and renovate the available objective evidence on the effectiveness of MBSR in improving anxiety, depression, perceived stress, sleep quality, mindfulness, self-kindness, social function, subjective well-being, and physical health among university students. The research was not registered in the International Prospective Registry of Systematic Reviews (PROSPERO).

## 2. Methods

### 2.1. Eligibility Criteria

Randomized controlled trials (RCTs) relating to mindfulness and stress reduction were included in this study. The included RCTs were limited to those published in English and that met the following criteria: (1) Participants were aged 18 years or older and (2) participants were university students who were enrolled in higher education institutions at the time of the trial. In the included studies, participants took part in the experiments voluntarily and met the criteria for either an MBSR intervention group or a control group (wait-list, no treatment, health education, and relaxation, etc.). The primary outcome index in the included RCTs included mental health problems and related outcomes that are common among university students (perceived stress, negative emotion, mindfulness, self-kindness, social function, subjective well-being, sleep problems, etc.). The secondary outcome index was physical well-being.

The exclusion criteria were as follows: (1) Participants were not registered university students; (2) their bodily functions could not handle MBSR therapy; (3) participants were diagnosed with mental disorders according to the classification of mental disorders, such as schizophrenia, major depression, panic disorders, or personality disorder; (4) participants were currently using psychoactive medication; (5) participants were currently undergoing individual psychotherapy or group intervention programs; and (6) participants suffered from cardiovascular diseases such as hypertension or arrhythmia. Participants were also excluded if they underwent any type of therapy or coaching during the study period.

### 2.2. Data Sources and Searches

Studies were identified by searching databases, scanning reference lists, and consulting with MBSR experts. The searched databases included PubMed (1966 to November 2023), EMBASE (1974 to November 2023), the Cochrane Library (issue 4 through 2023), ERIC (1907 to November 2023), the Web of Science (1974 to November 2023), Scopus, Google Scholar, and ProQuest. We used the following search terms: (“university students” [MeSH terms] OR “college students” [MeSH terms] OR “undergraduate” [MeSH terms] OR “academician” [MeSH terms] OR “graduate student” [MeSH terms] AND (“mindfulness-based stress reduction” [MeSH terms] OR “mindfulness-based stress reduction” [title/abstract] OR “mindfulness-meditation” [MeSH terms] OR “mindfulness-meditation” [title/abstract] OR “mind body therapies” [MeSH terms] OR “mind body therapies” [title/abstract] OR “mindfulness-based cognitive therapy” [MeSH Terms] OR “mindfulness-based cognitive therapy” [title/abstract] OR (“MBCT” [MeSH terms] OR “MBCT” [title/abstract]) OR (“mindfulness-based stress reduction” [MeSH Terms] OR “mindfulness-based stress reduction” [title/abstract]) AND (random^*∗*^ OR “clinical trials as topic” [Mesh] OR “clinical trial” [publication type]) (see Table 1 at the end of this manuscript for details).

### 2.3. Study Selection

Duplicate results were removed, and abstracts were screened using NoteExpress (v. 3.9). Two reviewers independently selected and agreed upon the abstracts to be excluded. Two of the authors reviewed the full texts of the included abstracts and discussed and agreed upon their eligibility decisions. Eligible studies addressed the beneficial stress relief and mental health outcomes of MBSR interventions among university students. Following this procedure, the selection process was conducted independently by two other authors, and any disagreements were discussed with a third reviewer if necessary. The data were extracted from the included studies, following the PRISMA checklist. These data mainly comprised the basic characteristics of the studies, characteristics of the study populations (sample size, age, and type of university student), intervention measures (type and specific protocol of the MBSR intervention, duration, and frequency), control measures, and outcome indicators. When the outcome indicators were described by the median and interquartile interval, they were converted to means and standard deviations as per the quantile estimation method.

### 2.4. Data Abstraction and Assessment of the Risk of Bias

A systematic review was performed following the Cochrane Handbook version 5.2.0 [18]. Three reviewers independently extracted the following information regarding each RCT: author name, publication time, mean age of participants, sample size, basic treatment regimen, control group settings, and outcome measures. The quality of the included studies was evaluated using the risk bias assessment tool provided by the Cochrane Collaboration [19]. Three reviewers independently assessed the methodological quality of the included studies. Two investigators then cross-checked the information, and any disagreements were resolved with a third party. The risk of publication bias across the included studies was examined using a funnel plot [20]. The systematic review and meta-analysis reporting were carried out in accordance with the PRISMA guidelines [21].

### 2.5. Data Analysis

Data were recorded using Excel 2010, and Stata software (version 10.0) was used for data processing. Statistical analysis was performed on the extracted data using standardized mean differences (SMDs). The effect sizes were expressed as 95% confidence intervals, and the test level was*α* = 0.05. Meta-analysis statistical models were weighted according to the combined effect size. A chi-square test was conducted to analyze heterogeneity, and *I*^2^ was used to evaluate the magnitude of heterogeneity. When *I*^2^ < 50%, a fixed-effects model was used to estimate the combined effect size. If *I*^2^ > 50%, the possible sources of heterogeneity were analyzed, and if the source of heterogeneity remained unidentified and unaddressed, a random-effects model was used to estimate the combined effect size.

The outcome indicators of the included studies were combined for effect size analysis, 95% confidence intervals of the overall parameters were employed for estimation, and the *U* test hypothesis was used. *p* < 0.05 indicated that the difference in the combined effect size of the included studies was statistically significant. The meta-analysis forest plot was performed using Stata (version 10.0, Stata Corp., College Station, TX, USA) [22] and Review Manager (RevMan, version 5.3) [19].

### 2.6. Grading of the Evidence

The international Grading of Recommendations, Assessment, Development, and Evaluation (GRADE) tool (GRADEpro GDT software, McMaster University, Canada, 2015) [23] was employed to assess the quality of evidence for the outcome indicators in the included studies. According to this tool, four factors can reduce the quality of the evidence presented: study limitations, inconsistent findings, indirect findings, and imprecise findings. The quality of the evidence presented in all the RCTs included in the present study was evaluated as moderate or high.

## 3. Results

### 3.1. Description of Studies

**Figure 1:**
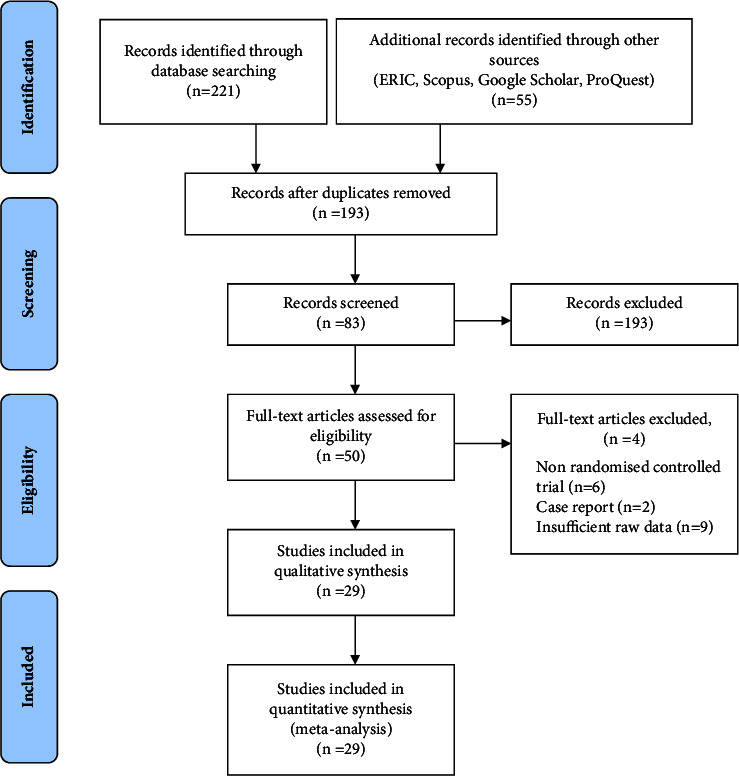
Flowchart of the literature search.

A total of 276 studies items were initially obtained through the database search. Using NoteExpress, 193 deduplicated studies were obtained, and the remaining 220 items were obtained. After reviewing their titles and abstracts, 135 studies that were of incompatible research types were excluded. A total of 13 articles were further excluded after reading the full text, and the remaining 29 studies [24–53] were retained for data extraction. The baselines of the trials were comparable. The literature search process and results are shown in Figure 1.

### 3.2. Study Characteristics

Sixteen of the RCTs were conducted in the United States [25–27, 29–33, 36, 37, 40, 43, 47, 48, 53], Canada [28], and Europe [26, 35, 38, 42, 44, 46, 51, 52]. One was conducted in South Korea [41], and three were conducted in China [33, 49, 50]. The participants' ages ranged from 18 to 30 years.

The inclusion criteria for 24 studies were references to previously published literature or online mindfulness programs [24–27, 30, 32–37, 39–44, 46–48, 50, 51, 53]. One was from the British Association of Mindfulness-Based Approaches 2020 [28], and the rest were from the standard Kabat-Zinn MBSR program [31, 38, 45, 49].

The intensities of the MBSR protocols of the RCTs varied, ranging from twice weekly for 20 min to 1.5 hours each. Treatment durations ranged from 2 to 10 weeks. MBSR prescriptions differed depending on session timeline, content, and frequency. Indications of MBSR mainly focused on mood regulation and an increase in participants' levels of satisfaction with their quality of life, self-acceptance, social function, subjective well-being, physical health, and sleep quality (Tables 2 and 3).

### 3.3. Methodological Quality of the Included Studies

**Figure 2:**
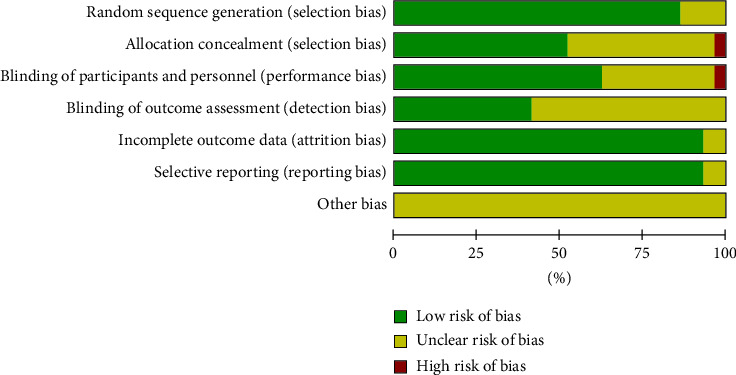
Methodological quality of the included studies.

Of the 29 RCTs included, none fulfilled all the methodological criteria. Various forms of randomization procedures were adopted in the studies. In 22 of the included RCTs, blocks were concealed and sequences were stored in sealed, opaque, numbered envelopes, or another concealed allocation protocol was used [25–35, 37, 41, 42, 44–53]. Five studies reported the blinding of the research assistant and the participants [28, 33, 34, 37, 53], fourteen reported the blinding of the participants [26, 27, 39, 41, 42, 44–48, 50–52], two reported the blinding of the participants and the therapists [25, 39], and one reported the blinding of the investigators, statistician, and participants and/or the university students [30] (Figure 2 and Table 3).

### 3.4. Outcome Analysis

**Figure 3:**
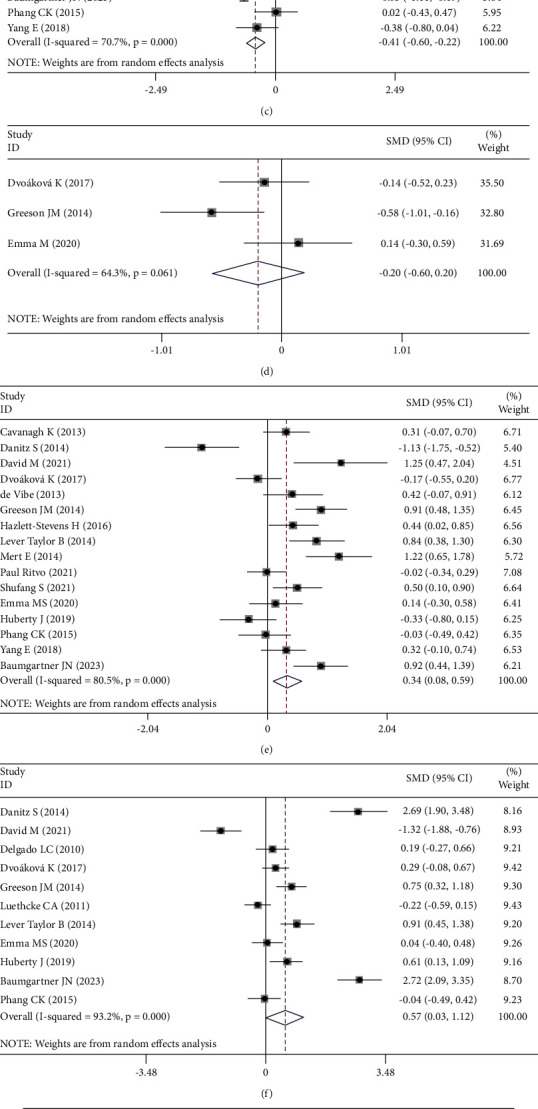
A forest plot of the effects of the group mindfulness-based stress reduction therapies on treatment-related side effects. The width of the horizontal lines represents the 95% confidence intervals (CIs) of the individual studies, and the squares represent the proportional weight of each study. The diamonds represent the pooled odds ratio and 95% CI. (a) Anxious, (b) depression, (c) perceived stress, (d) sleep quality, (e) mindfulness, (f) self-kindness, (g) social function, (h) subjective well-being, and (i) physical health.

Compared with control therapies, the pooled results suggested that MBSR showed significant effects in reducing anxiety (SMD = −0.29; 95% CI: −0.49 to −0.09), depression (SMD = −0.32; 95% CI: −0.62 to −0.02), and perceived stress (SMD = −0.41; 95% CI: −0.60 to −0.29) and improving mindfulness (SMD = 0.34; 95% CI: 0.08 to 0.59), self-kindness (SMD = 0.57; 95% CI: 0.30 to 1.12), and physical health (SMD = −0.59; 95% CI: −1.14 to −0.04). No significant differences were observed in sleep quality (SMD = −0.20; 95% CI: −0.06 to 0.20), social function (SMD = −0.71; 95% CI: −2.40 to 0.97), or subjective well-being (SMD = 0.07; 95% CI: −0.18 to 0.32). No adverse reactions were reported in any of the randomized controlled trials (Figure 3 and Table 4).

Heterogeneity was present in the comparison of studies on anxiety (*I*^2^ = 72.4%), depression (*I*^2^ = 86%), perceived stress (*I*^2^ = 70.7%), sleep quality (*I*^2^ = 63.4%), mindfulness (*I*^2^ = 80.5%), self-kindness (*I*^2^ = 93.2%), social function (*I*^2^ = 98.1%), subjective well-being (*I*^2^ = 78.5%), and physical health (*I*^2^ = 86%). A meta-regression revealed that the effects of age, inclusion criteria, indications, and duration did not explain this heterogeneity. The meta-regression results showed that the effect of the duration of the MBSR therapy on anxiety (*p* ≤ 0.02), perceived stress (*p* ≤ 0.01), and subjective well-being (*p* ≤ 0.001) and the effect of the type of control group on depression (*p* ≤ 0.01) partly explained the heterogeneity. Age, recruitment, MBSR duration, control group, and the regional differences of participants were not the sources of heterogeneity for the effects on sleep quality, mindfulness, self-kindness, social function, and physical health (all *p* ≥ 0.05) (Table 5).

**Figure 4:**
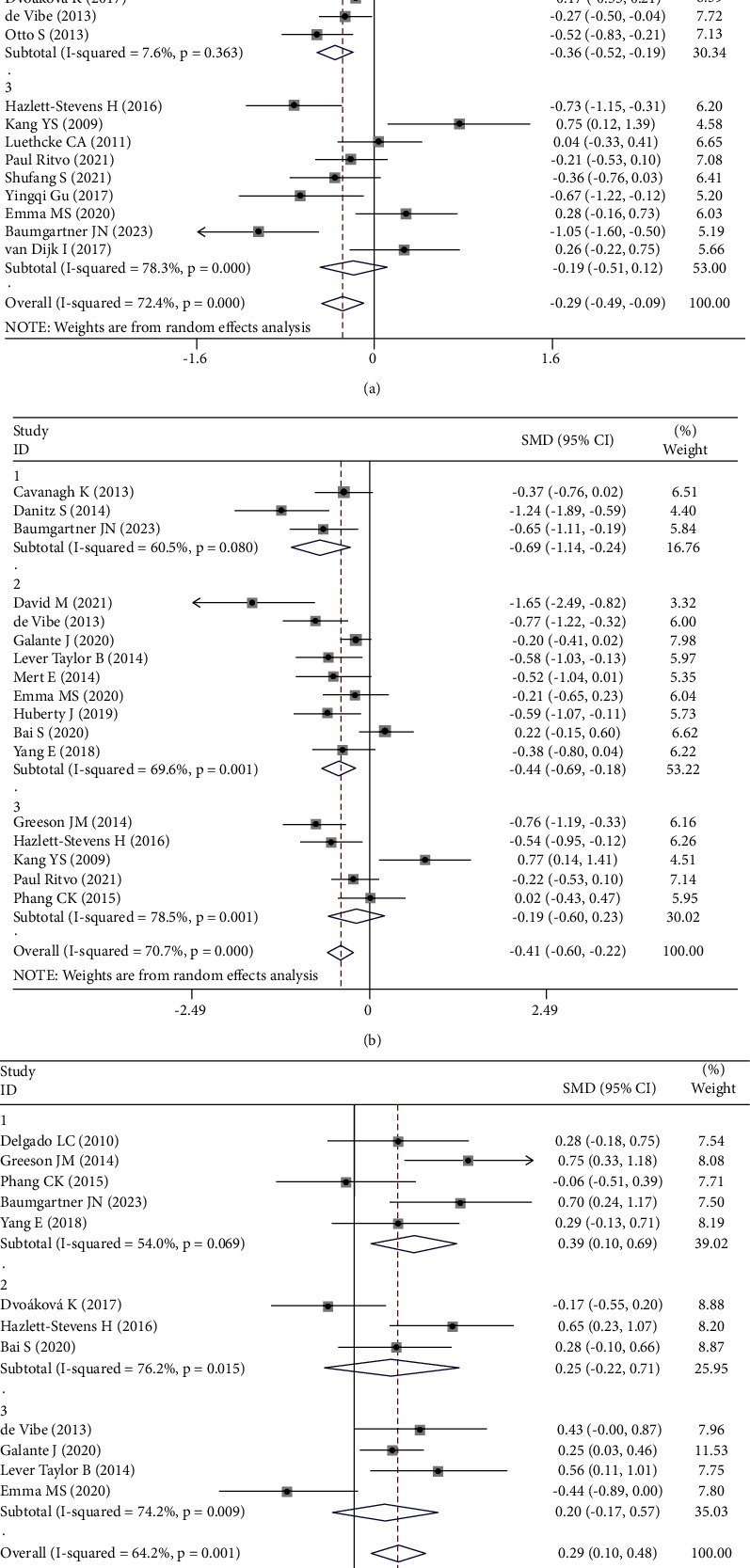
Forest plot of the effect sizes of subgroup analysis of mindfulness-based stress reduction therapies. The width of the horizontal line represents the 95% CI of the individual studies, and the square proportional represents the weight of each study. The diamond represents the pooled OR and 95% CI. (a) Subgroups for anxious, (b) subgroups for perceived stress, and (c) subgroups for subjective well-being.

Further subgroup analyses were conducted to examine the effect of different control groups (no treatment, health education, and social support) on depression. Subgroup analyses were also conducted to examine the effect of MBSR duration (less than 2 weeks, 4–6 weeks, and over 8 weeks) on anxiety, perceived stress, and subjective well-being (Figure 4).

**Figure 5:**
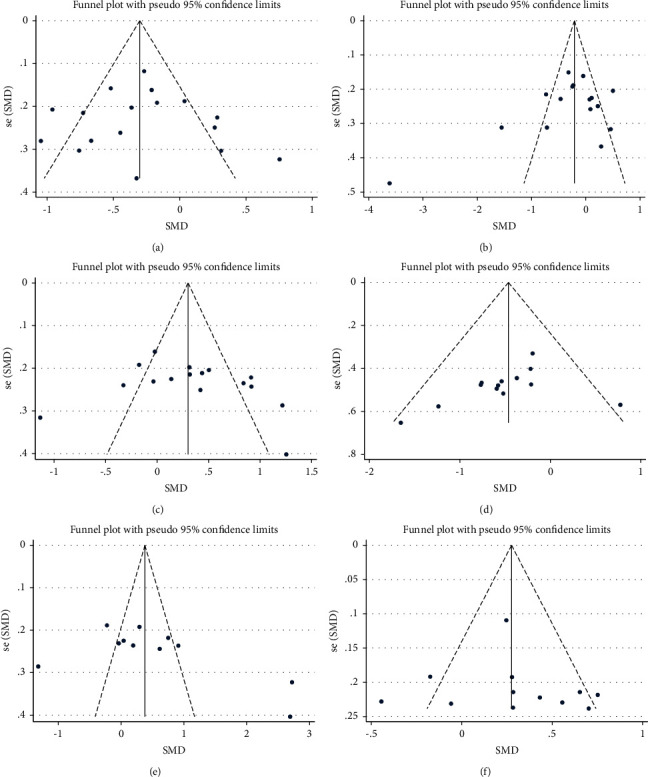
Funnel plot displaying the probable publication bias in the overall estimated pooled effect size of MBSR on primary outcomes. (a) Anxiety, (b) depression, (c) mindfulness, (d) perceived stress, (e) self-kindness, and (f) subjective well-being.

The subgroup analysis showed that different control groups had different effects on the outcome indicators of depression. The intervention effect of the blank control group was the most pronounced (SMD = −0.69; 95% CI: −1.43 to 0.04). The subgroup analysis also demonstrated that MBSR programs of 2–4 weeks or more than 6–8 weeks improved the outcome indicators of anxiety (SMD = −0.29; 95% CI: −0.49 to 0.09), stress perception (SMD = −0.41; 95% CI: −0.60 to −0.22), and subjective well-being (SMD = 0.29; 95% CI: 0.10 to 0.48) (Figure 4). Egger's test showed that there was no indication of publication bias for any of the outcomes (Figure 5).

### 3.5. GRADE Assessment

The quality of the evidence presented in the reviewed studies was assessed with the GRADEpro GDT software tool. Table 3 shows a summary of the overall assessment of the quality of evidence regarding the effect of MBSR on the outcome control measures. The quality classification for the evidence for each variable was as follows: anxiety: moderate; depression: moderate; perceived stress: moderate; sleep quality: moderate; mindfulness: moderate; self-kindness: moderate; social function: moderate; subjective well-being: moderate; and physical health: moderate. The evidence for sleep quality and physical health was downgraded for indirectness (Table 6).

## 4. Discussion

Mindfulness decompression interventions are considered one of the most popular forms of psychological intervention [54]. Well-designed related studies on the efficacy of mindfulness for mental disorders have been conducted since Dr. Jon Kabat-Zinn founded mindfulness decompression therapy in 1982 [55]. Mindfulness decompression therapy continues to be gradually recognized in an increasing number of professional fields [56, 57]. In this type of therapy, participants are guided to reflect on their personality and mental tendencies and release internal conflicts and stressors. Participants also explore the potential of self-orientation in order to achieve meditation and intellectual flow, stabilize and adjust their emotions, reduce or eliminate negative feelings, and achieve a mild healing effect through physical and mental balance [58].

Existing research generally recognizes that mindfulness decompression psychological interventions can promote communication, enhance individual self-esteem, relieve emotions, and improve behavior. However, it is difficult to quantify the effect of such interventions; thus, this represents an important direction for researchers in related fields as they continue to expand the evidence base supporting mindfulness decompression interventions [59, 60].

Our meta-analysis showed that there was heterogeneity between groups in relation to the effects of the duration of MBSR therapy on anxiety, depression, perceived stress, and subjective well-being. In our meta-analysis and systematic review, the subgroup analysis demonstrated that timed MBSR therapy intervention protocols had clear benefits for anxiety, depression, and perceived stress. The subgroup analysis also revealed a linear relationship between the duration of the intervention and antinegative emotions and improved self-positive acceptance concepts, suggesting that relatively short interventions (e.g., two weeks) had a potential impact on the management of negative emotions and buffered stress. As long as the psychological intervention of mindfulness therapy was prescribed, the intervention of the study participants was effective. Due to its design, the current study could not determine the results of subsequent long-term follow-up.

The subgroup analysis of depression control groups showed that the degree of remission of depressive symptoms in the no-treatment control group was significantly lower than that in the support and health education control groups. In the subgroup analyses of the effects of interventions on anxiety, perceived stress, and mindfulness, different intervention durations showed a mild improvement effect. MBSR attaches importance to an individual's physical condition, flexibility, and perception of physical and mental activities and attaches more importance to the adjustment and adaptation of their overall function. These factors reflect the difficulty and complexity of MBSR research, while also presenting opportunities and challenges for the integration of general practice via the existing MBSR research methodology.

The subgroup analysis also revealed that the effects of MBSR interventions on depression outcome indicators were sensitive to the type of control group. As the MBSR intervention had a minimal impact on students in the control groups, it may be that the type of control group affected the initial positive findings.

The evaluation of the effect size of the control groups in the RCTs included in this review highlighted that when there was an imbalance in the baseline of control participants, the statistical data may be unstable, masked, or magnified. Each MBSR RCT adopted a different study design and implemented control groups differently. For some variables, the choice of the control group had far more influence than the MBSR protocol on the research outcomes: This deficiency was partly due to the design of the control groups.

The meta-regression showed that there was heterogeneity between the length of the MBSR intervention and the control group, and the subgroup analysis showed that whether the effect of MBSR was pronounced or not was closely related to the duration of MBSR and the control groups. Practice heterogeneity and methodological heterogeneity are the main sources of heterogeneity in meta-analyses. Consequently, appropriate interpretation and analysis methods must be adopted to ensure the reliability of meta-analysis results.

The prevalence of physical function and sleep problems among university students is relatively high, and sleep problems tend to last for years after the end of treatment [61, 62]. University students' psychological and social adaptation difficulties may reflect the negative consequences of poor physical health and sleep quality. Previous studies have revealed that more than half (52.7%) of university students sleep 6 to 8 hours per night, 37% sleep less than 6 hours, and 40% go to sleep after 2 am [63–65]. Our meta-analysis observed no significant difference in improvement in sleep disorders following MBSR interventions. The included studies evaluated sleep quality after intervention and at 4–12 weeks postintervention. In the studies on sleep quality in our meta-analysis, the average follow-up time was 6.67 weeks, which was likely not long enough. Future RCTs examining the effects of MBSR intervention on sleep quality and physical health should standardize follow-up times and aim to establish the content validity of the domains of sleep quality and physical health that are assessed.

Our meta-analysis suggests that mindfulness decompression psychological intervention is conducive to the psychological and behavioral health of university students. The positive results of this type of intervention are manifested in the release of negative emotions, such as anxiety, depression, and perceived stress. MBSR interventions also show a positive effect on the establishment of self-confidence among university students. The blinding of participants, personnel, and outcome assessors was performed in most studies in our meta-analysis and systematic evaluation. However, defects in these studies' research methods limit the interpretation of the effectiveness of these findings. The sample sizes of the eligible studies were small, and their methods of estimating these sample sizes were unclear. Globally, there is an overall lack of high-quality random comparison studies of MBSR interventions involving university students.

Not all the included studies adopted the standard design of mindfulness decompression psychological intervention programs. They also did not all describe (1) whether the implementer had received professional mindfulness decompression psychological intervention training; (2) the professional knowledge of the mindfulness decompression psychological intervention practitioners; or (3) the frequency, intensity, content, or duration of the mindfulness decompression psychological intervention. Furthermore, the settings of the control groups and the heterogeneity of the basic physical and sociological characteristics of the university student participants affect the reliability of the conclusions drawn in these studies. Such inconsistencies and imprecision were major reasons for downgrading the accuracy and quality of the evidence recommending the use of MBSR therapy in research involving university students. They also show underlying insignificant psychological efficacy.

There were further serious concerns related to four GRADE domains (risk of bias, inconsistency, indirectness, and imprecision) across the three studies that measured MBSR. As such, the certainty of the evidence that MBSR promotes well-being through improved sleep quality was very low. We identified 29 studies that employed a range of mindfulness meditation methods, of which all but nine were published within the past decade. Nevertheless, there were relative consistency concerns related to two or more GRADE domains (risk of bias, inconsistency, and indirectness or imprecision) across the studies, implying a moderate certainty in the evidence that MBSR reduces psychological distress and promotes well-being among university students.

In addition, the results indicate that it is important that the mental health gains generated by mindfulness stress reduction practices among college students are sustained over time. After some time, students may become more aware of the improvements in their well-being and mental health owing to MBSR interventions—they will feel better about themselves and realize that improvements in their physical health, stress levels, and well-being were gained through meditation training. Thus, though the effects of MBSR training can be validated immediately after an intervention, they may not yet be consolidated to the point that recipients consciously recognize all of these effects. The effects of meditation practice are gentle, accumulate slowly, and become more pronounced over time as students practice what they have learned. Furthermore, among university students, medical students tend to better perceive the benefits of training when exposed to stressful situations. When the knowledge learned in training can be put into practice in challenging specific situations, students can evaluate the effectiveness of meditation practices as a stress management tool; however, a nonmedical intervention group of students is likely to be unaffected by these infrequent events.

It should also be noted that despite the widespread adoption of the blind method, assessing perceived improvement in students' psychological distress and well-being is a complex issue because the assessments were conducted by students themselves. As such, they are subjective, even when considering the heterogeneity of the questionnaire tools used to validate such assessments. It should also be considered that in the psychological context, no measure can fully define the complexity of behavior in the real world, and measures of abstract mental constructions can only provide an indirect assessment. The psychological questionnaire instruments in the included studies involved self-reporting by students; therefore, the potential bias caused by self-reporting, for example, social desirability bias, could not be controlled for. However, given that the instruments used to measure quality of life in the analyzed studies were all psychometrically sound, this limitation is unlikely to be serious. Egger's test funnel plot ruled out the probability of publication bias.

This meta-analysis has some limitations. Owing to our research design, we only included RCTs that were published in English; therefore, there is a possibility that some important, high-quality findings that were not published in English may not have been included. Moreover, the quality of the included RCTs was generally adequate; however, though there was no indication of publication bias, the low number of studies decreases the reliability of this result. Given the small sample size of the included RCTs, high-quality, large-scale clinical studies are needed to provide further evidence in the future. Multicenter studies with large samples are required to explore standardized intervention protocols, MBSR practitioners' professional knowledge, the inconsistent design of MBSR protocols (frequency, intensity, content, and duration), the heterogeneity of control groups, and university students' physical and sociological characteristics. These factors may greatly affect the clinical significance of MBSR in terms of the average effect of the intervention on university students' overall quality of life. These clinical and methodological factors may, consequently, have affected the reliability of the conclusions drawn in extant research.

The effects and influences of mindfulness decompression psychological interventions on psychological outcomes, quality of life, emotional habits, and behavioral disorder symptoms among university students remain unclear. Intervention elements, such as the environment of mindfulness decompression psychological intervention in colleges and universities, university students' mindfulness communication and expression abilities, the level of language communication, and the standardized implementation guidelines for different intervention subtypes require further research. Future studies should aim to use larger samples, different forms of mindfulness, and long-term follow-up. They should also aim to obtain detailed information on sociodemographic variables and the intermediary and regulatory variables of mindfulness decompression psychological interventions. All these factors need to be explored further.

## Figures and Tables

**Table 1:** Systematic review search strategy: research terms used to identify RCT of mindfulness-based stress reduction in those databases.

PubMed	(“Students, University”[Mesh] or (university students) or (Student, college) or (College Student) or (Undergraduate students) or (undergraduate students) or (undergraduate education)) AND (“Mindfulness”[Mesh] or (Mindfulness meditation) or (Kabat-Zinn protocol) or (Kabat-Zinn programme) or (mindfulness-based stress reduction) or (mind-body skill training) or (MBSR) or (Mindfulness) or (mindfulness) AND (“Randomized controlled trial”[Mesh] or (RCT) or (randomized controlled trial)

EMBASE	(“University student”/exp OR “university student” OR “university students”/exp OR “college students” OR “college student” OR “undergraduate education”/exp OR “undergraduate education” OR “college students”/exp OR “college student”) AND (“mindfulness”/exp OR “mindfulness” OR “mindfulness-based stress reduction”/exp OR “mindfulness-based stress reduction” OR “mindfulness training”/exp OR “mindfulness training” OR “mindfulness meditation”/exp OR “mindfulness meditation” OR “mindfulness-based stress reduction program”/exp OR “mindfulness-based stress reduction program”) or (“Kabat-Zinn protocol”/exp OR “Kabat-Zinn protocol” OR “Kabat-Zinn programme” OR MBSR'/exp OR “MBSR”) AND (“Randomized controlled trial”/exp OR “Randomized controlled trial” OR (“Randomized controlled trials”/expor (“Randomized controlled trials” or (“RCT”/exp OR “RCT”)

Web of Science	((((((((((University students) or (university students) or (university student) or (university student) or (College students) or (College students) or (College student) or (College student) or (Undergraduate students) or (undergraduate students) or (undergraduate student)))))))))) or (undergraduate education) AND (Mindfulness) or (Mindfulness meditation) or ((((((((Kabat-Zinn programme) or (Kabat-Zinn protocol) or (Kabat-Zinn) or (mindfulness-based stress reduction) or (mind-body skill training) or (MBSR)))))))) AND (((Randomized controlled trial) or (Randomized controlled trials) or (randomized controlled trials) or (RCT)))

PsycINFO (formerly PsychLit)	((((((((((University students) or (university students) or (university student) or (university student) or (College students) or (College students) or (College student) or (College student) or (Undergraduate students) or (undergraduate students) or (undergraduate student)))))))))) or (undergraduate education) AND (Mindfulness) or (Mindfulness meditation) or ((((((((Kabat-Zinn programme) or (Kabat-Zinn protocol) or (Kabat-Zinn) or (mindfulness-based stress reduction) or (mind-body skill training) or (MBSR)))))))) AND (((Randomized controlled trial) or (Randomized controlled trials) or (randomized controlled trials) or (RCT)))

Ovid MEDLINE	#1 university/college students: ti, ab, kw
	#2 MeSH descriptor: [university/college students] explode all trees
	#3 Undergraduate students: ti, ab, kw
	#4 MeSH descriptor: [Undergraduate students] explode all trees
	#5 #1 or #2 or #3 or #4
	#6 MeSH descriptor: [mindfulness-based stress reduction] explode all trees
	#7 mindfulness-based stress reduction: ti, ab, kw or MBSR: ti, ab, kw or Mindfulness meditation: ti, ab, kw or Mindfulness: ti, ab, kw or Kabat-Zinn programme: ti, ab, kw or Kabat-Zinn protocol: ti, ab, kw or Kabat-Zinn: ti, ab, kw
	#8 #1 or #2 or #3 or #4
	#9 Randomized controlled trial: ti, ab, kw
	#10 MeSH descriptor: [randomized controlled trial] explode all trees
	#11 Randomized controlled trial: ti, ab, kw or randomized controlled trial: ti, ab, kw or RCT
	#12 #5 or #8 or #11

ERIC	((((((((((University students) or (university students) or (university student) or (university student) or (College students) or (College students) or (College student) or (College student) or (Undergraduate students) or (undergraduate students) or (undergraduate student)))))))))) or (undergraduate education) AND (Mindfulness) or (Mindfulness meditation) or ((((((((Kabat-Zinn programme) or (Kabat-Zinn protocol) or (Kabat-Zinn) or (mindfulness-based stress reduction) or (mind-body skill training) or (MBSR)))))))) AND (((Randomized controlled trial) or (Randomized controlled trials) or (randomized controlled trials) or (RCT)))

Scopus	#1 university/college students: ti, ab, kw
	#2 MeSH descriptor: [university/college students] explode all trees
	#3 Undergraduate students: ti, ab, kw
	#4 MeSH descriptor: [Undergraduate students] explode all trees
	#5 #1 or #2 or #3 or #4
	#6 MeSH descriptor: [mindfulness-based stress reduction] explode all trees
	#7 mindfulness-based stress reduction: ti, ab, kw or MBSR: ti, ab, kw or Mindfulness meditation: ti, ab, kw or Mindfulness: ti, ab, kw or Kabat-Zinn programme: ti, ab, kw or Kabat-Zinn protocol: ti, ab, kw or Kabat-Zinn: ti, ab, kw
	#8 #1 or #2 or #3 or #4
	#9 Randomized controlled trial: ti, ab, kw
	#10 MeSH descriptor: [randomized controlled trial] explode all trees
	#11 Randomized controlled trial: ti, ab, kw or randomized controlled trial: ti, ab, kw or RCT
	#12 #5 or #8 or #11

Google Scholar and ProQuest	University students, university student, college students, college student or undergraduate students or undergraduate education and mindfulness or Mindfulness meditation or Kabat-Zinn programme or Kabat-Zinn protocol or Kabat-Zinn or mindfulness-based stress reduction or mind-body skill training or MBSR

Cochrane Library	Cochrane Library 504 studies
	#1 MeSH descriptor: [Students, General] explode all trees
	#2 University General
	#3 university general
	#4 (Student, College)
	#5 (student, college)
	#6 MeSH descriptor: [Undergraduate Students] explode all trees
	#7 (Undergraduate)
	#8 (undergraduate students)
	#9 (undergraduate education)
	#10 #1 or #2 or #3 or #4 or #5 or #6 or #7 or #8 or #9
	#11 MeSH descriptor: [Mindfulness] explode all trees
	#12 (Mindfulness meditation)
	#13 (Kabat-Zinn protocol)
	#14 (MBSR programme)
	#15 (mindfulness-based stress reduction)
	#16 (mind-body skill training)
	#17 (Mindfulness)
	#18 (MBSR)
	#19 (Kabat-Zinn)
	#20 (Mindfulness-Based Cognitive Therapy)
	#21 #10 or #11 or #12 or #13 or #14 or #15 or #16 or #17 or #18 or #19 or #20
	#22 (Randomized controlled trial)
	#23 (RCT)
	#24 (randomized controlled trial)
	#25 #22 or #23 or #24
	#26 #10 AND #21 AND #25

**Table 2:** Characteristics of the included studies.

Authors/year/country	Type/recruitment/S general university student sample	No. of student sample (MBSR/control group)	Mean age of MBSR group	Mean age of control group	Inclusion criterion	MBSR protocol	Control group	Outcome measures/results
Bai et al. 2020 [24] USA	First-year college students	55/54	18 and above	18 and above	Previous published references	80-minute, 8 sessions, 6 week	Wait-list	Significant reduction in stress (DISE, *p* < 0.05); no significant improvement in negative emotion (NEI, *p* > 0.05), rumination (RRQ, *p* > 0.05)
Baumgartner and Schneider 2023 [25] USA	University general student sample	29/29	18–23	18–23	Previous published references	2-week period, 10-min mindfulness practice	Wait-list	No significant reduction in stress (PSS, *p* < 0.05); significant improvement (GPA, *p* < 0.05)
Cavanagh et al. 2013 [33] USA	University general student sample	54/50	25.28 ± 6.85	24.08 ± 5.98	Previous published references	Online MBSR, 10-min daily, 2 weeks	Wait-list	Significant reduction in depression (FFMQ, *p* ≤ 0.001), (PSS, *p* < 0.05), (PHQ-4, *p* < 0.05)
Chen et al. 2013 [34] USA	University first-year nursing students	30/30	Unclear	Unclear	Previous published references	7 consecutive days mindfulness meditation, 30-min each time	Health education	Significant reduction in anxiety (SAS, *p* < 0.05), depression (SDS, *p* < 0.05), and autonomic nervous system function improved (SBP, *p* < 0.05), (DBP, *p* < 0.05)
Danitz and Orsillo 2014 [30] USA	University first-year undergraduates and first-year law students	19/30	18 and above	18 and above	Previous published references	1.5-hr workshop, a 2-week period, 10-min mindfulness practice	Wait-list	Significant reduction in depression (DASS, *p* < 0.05); acceptance (PHL-MS, VLQ, *p* < 0.05); no significant reduction in anxiety (SAS, *p* > 0.05)
Martínez-Rubio et al. 2021 [35] UK	University undergraduate psychology students	15/15	22.08 ± 3.65	22.5 ± 4.64	Previous published references	6 MBSR program topic of 90-min sessions weekly per week, 6 weeks	Wait-list	Significant reduction in stress and psychological distress (PSS, GHQ-12, *p* < 0.05), (FFMQ, *p* < 0.05), and self-compassion (SCS-SF, *p* < 0.05), (AAQ, *p* < 0.05)
Delgado et al. 2010 [36] USA	University female students	36/36	18–24	18–24	Previous published references	Mindfulness, and progressive muscle relaxation, weekly 1-hr group sessions, 5 weeks	Relaxation	Significant improvement in emotional and physiological regulatory mechanisms (STAI, *p* < 0.05), (BDI, *p* < 0.05), (PANAS, *p* < 0.05), (SHC, <0.05), (TMMS, *p* < 0.05), heart rate (*p* < 0.05)
Dvořáková et al. 2017 [37] USA	University first-year undergraduate students	55/54	18.2 ± 0.4	18.2 ± 0.4	Previous published references	8 sessions over 6 weeks, each session 80-min	Wait-list	Significant increase in life satisfaction (SCC, *p* < 0.05), (SWL, *p* < 0.05), (MAAS, *p* < 0.05); significant decrease in depression (GHQ, *p* < 0.05) and anxiety (GAD, *p* < 0.05)
Vibe et al. 2013 [38] NOR	University medicine and psychology	26/43	23.8 ± (5.2)	23.6 ± 4.7	Kabat-Zinn MBSR programme	8 weeks, each sessions 2.5-hr	Wait-list	Significant reduction in mental distress (GHQ-12, *p* < 0.05), (MBI, *p* < 0.05), (PMSS, *p* < 0.05); significant improvement in subjective well-being (SWB, *p* < 0.05) and (FFMQ, *p* < 0.05)
Seppälä et al. 2020 [26] UK	University undergraduate students	34/47	18 and above	18 and above	Online mindfulness program	Twice per week for a total of 30-min, 8 weeks	No-treatment	Significant reduction in burnout (SIMB, *p* < 0.05), distress, anxiety, depression (MASQ, *p* < 0.05), stress (PSS, *p* < 0.05); significant improvement in life satisfaction (SWLS, *p* < 0.05), positive affect (PANAS, *p* < 0.05), gratitude (GQ-6, *p* < 0.05), self-compassion (SCS, *p* < 0.05), mindfulness (FFMQ, *p* < 0.05), and self-esteem (SISE, *p* < 0.05)
Galante et al. 2020 [32] USA	University undergraduate or postgraduate students	168/169	18 and above	18 and above	Previous published references	75–90-min MBSR, home practice 15–25-min/day, 8 weeks	Support	No significant improvement in distress (CORE-OM, *p* > 0.05); mental well-being (WEMWBS, *p* > 0.05)
Greeson et al. 2014 [31] USA	University undergraduate, graduate, and professional students	45/45	25.75 ± 6.84	24.76 ± 4.15	Kabat-Zinn MBSR programme	75-min classes, meditation 10 minutes daily, 4 weeks	Wait-list	Significantly reduce perceived stress (PSS, *p* > 0.05), increase self-compassion (CAMS-R, *p* > 0.05), (SCS, *p* < 0.05), gratitude (GQ-6, *p* > 0.05), and sleep quality (MOS SLP9, *p* > 0.05)
Gu et al. 2018 [49] CHA	Undergraduate, graduate, and professional students	45/45	25.4 ± 5.7	25.4 ± 5.7	Kabat-Zinn MBSR programme	75-minute classes first time, 10-min daily, 4 weeks	Wait-list	Significant reduce perceived stress (PSS, *p* < 0.05), sleep problems(MOS SLP9, *p* < 0.05), increase mindfulness (CAMS-R, *p* < 0.05), and self-compassion (SCS, *p* < 0.05)
Hazlett-Stevens and Oren 2016 [40] USA	University undergraduate students	28/26	20.21 ± 1.03	20.38 ± 1.02	Previous published references	1-hour MBSR sessions, 30 min of self-practice per day, 6 weeks	Wait-list	Significant reduction in depression, (BDI, *p* ≤ 0.001), anxiety (BAI, *p* < 0.05); significant improvement in self-compassion (FFMQ, *p* < 0.05)
Huberty and Oren 2019 [27] USA	University undergraduate students	47/45	22.1	22.1	Previous published references	1-week workbook content quiz, 10-week guided intervention meditation	No-treatment	Significant increased mindfulness (FFMQ, *p* < 0.05), quality of life (WHOQOL, *p* < 0.05); significant decreases in depression (DASS, *p* < 0.05), anxiety (PSS, *p* < 0.05), stress (PSWQ, *p* < 0.05)
Kang et al. 2009 [41] KOR	University female undergraduates	58/55	18.45 ± 0.78	18.45 ± 0.78	Previous published references	Nonjudgmental, and cognitive dissonance-based MBSR, 4 weeks	No-judgmental control	No significant improvement in body image dissatisfaction (BIAQ, *p* > 0.05), (BCQ, *p* > 0.05), (SBPS, *p* > 0.05), depression (BDI-II, *p* > 0.05), and eating disorders (EDE-Q, *p* > 0.05)
Kvillemo et al. 2016 [42] SWE	University undergraduate and postgraduate students	40/39	30.50 ± 10.78	26.67 ± 6.75	Previous published references	2-3 times a week, 8 weeks sessions MBCT	Wait-list	Significant reduction in depression (DASS, *p* < 0.05), anxiety (DASS, *p* < 0.05), stress (DASS, *p* < 0.05); significant increase in satisfaction with life (SWLS, *p* < 0.05), self-compassion (SCS, *p* < 0.05)
Luethcke et al. 2011 [43] USA	University female undergraduates	58/55	22.02 ± 5.52	24.18 ± 9.95	Previous published references	Videoconferences MBSR biweekly in three 20-min evening sessions, 8 weeks	No-judgmental control	Significant improvement in body image (BIAQ, *p* < 0.05), (BCQ, *p* < 0.05), (SBPS, *p* < 0.05), eating disorders (EDE-Q, *p* < 0.05); no significant improvement in depression (BDI, *p* > 0.05)
Lever Taylor et al. 2014 [44] UK	University nursing students	16/16	22.69 ± 1.49	22.25 ± 0.86	Previous published references	90-min per day for 8 weeks session MBSR	Health education	Significant reduction in anxiety (STAI, *p* < 0.05), stress (PWI-SF, *p* < 0.05); no significant improvement in depression (BDI, *p* > 0.05)
Erogul et al. 2014 [45] USA	University undergraduate students	46/44			Kabat-Zinn MBSR programme	30–45 minutes, 6 to 7 days per week, 8 weeks session MBSR	Wait-list	No significant increase in mental well-being (FFMQ, *p* > 0.05) and depression (SDS, *p* > 0.05)
Simonsson et al. 2013 [29] USA	University first-year medical students	28/30	23.5 ± 1.7	23.3 ± 1.4	Previous published references	75-min weekly at home and a half-day retreat in the last week, 6 months	No-treatment control	No significant increase in stress (PSS, *p* > 0.05), mental resilience (RS, *p* > 0.05), self-compassion (SCS, *p* > 0.05)
Ritvo et al. 2021 [28] CAN	University students	88/89	18 and above	18 and above	British Association of Mindfulness-Based Approaches 2020	20–30 minutes per day, 4 weeks	Wait-list	Significant reduction in anxiety (PROMIS, *p* < 0.05); no significant reduction in depression (PROMIS, *p* > 0.05)
Phang et al. 2015 [50] CHA	Medical students	37/38	21.14 ± 1.10	20.94 ± 1.17	Previous published references	5 week/day, thought-scan, body scan and kindness, home-gym (homework assignment)	Health education	Significant reduction in stress (GHQ, *p* < 0.05); significant improvement in subjective well-being (GSES, *p* > 0.05)
Recabarren et al. 2019 [46] CH	University students	76/78	22.02 ± 5.52	24.18 ± 9.95	Previous published references	Videoconferences MBSR biweekly in three 20-min evening sessions, 8 weeks	Wait-list	Significant reduction in anxiety (BAI, *p* < 0.05), mindfulness (FFMQ, *p* < 0.05); no significant reduction in depression (PHQ, *p* > 0.05), and stress (PSS, *p* > 0.05)
Sun et al. 2021 [47] USA	Undergraduate psychology university students	32/32	21.22 ± 2.27	21.47 ± 2.8	Mini-International Neuropsychiatric Interview	Cognitive behavioral mindfulness-based stress prevention, 2-h each sessions, 8 weeks	Wait-list	Significant reduction in depression (BDI, *p* < 0.05), anxiety (STAI, *p* < 0.05), pain (WHOQOL, *p* < 0.05), no significant improvement in self-efficacy (GSES, *p* > 0.05), coherence (SOC, *p* > 0.05), self-compassion (SCS, *p* > 0.05), and social support (MSPSS, *p* > 0.05)
Song and Lindquist 2015 [48] USA	University students	52/47	18 or older	18 and above	Previous published references	40-min each, every day, 4 weeks	Support	Significant increase in mindfulness (MAAS, *p* < 0.05); no significant improvement in depression (PHQ, *p* > 0.05) and anxiety (GAD, *p* > 0.05)
van Dijk et al. 2017 [51] NL	Medical students	83/84	23.7 ± 1.91	23.3 ± 1.77	Previous published references	10–20 min of daily mindfulness meditation for 30 days	No-treatment	Significant increase in mindfulness (MAAS, *p* < 0.05); significant improvement in stress (PSS, *p* > 0.05) and general health (GHQ, *p* > 0.05); significant decreases in self-efficacy (GSES, *p* > 0.05)
Yang et al. 2018 [53] USA	Medical students	45/43	21	21	Previous published references	10–20 min of daily mindfulness meditation for 30 days	No-treatment	Significant increase in mindfulness (FFMQ, *p* < 0.05) and mindfulness (FFMQ, *p* < 0.05); significant decreases in stress (PSS, *p* < 0.05)
Gu et al. 2017 [39] CHA	University nursing students	21/23	19–24	19–24	Previous published references	2 hr every day, 8 weeks	Wait-list	Significant decreases in depression (DASS, *p* < 0.05), anxiety (DASS, *p* < 0.05), stress (DASS, *p* < 0.05) and increase in mindfulness (MAAS, *p* < 0.05)
Warnecke et al. 2011 [52] AUS	Medical students	31/34	23.92 ± 3.2	23.92 ± 3.2	Previous published references	CD contained 30 min of spoken guided mindfulness practice, every day, 8 weeks	No-treatment	Significant decreases in depression (PSS, *p* < 0.05), anxiety (DASS, *p* < 0.05), stress (PSS, *p* < 0.05)

Young Adult Alcohol Problems Screening Test, YAAPST; Acceptance and Action Questionnaire, AAQ; Anxiety Sensitivity Index, ASI; Beck Anxiety Inventory, BAI; Beck Depression Inventory, BDI; blood pressure, BP; Body Image Avoidance Questionnaire, BIAQ; Body Checking Questionnaire, BCQ; Conners' Adult ADHD Self-Rating Scale, CAARS; Clinical Outcomes in Routine Evaluation Outcome Measure, CORE-OM; Cognitive and Affective Mindfulness Scale-Revised, CAMS-R; Eating Disorder Examination Questionnaire, EDE-Q; Depression Anxiety and Stress Scales, DASS; diastolic blood pressure, DBP; Daily Inventory of Stressful Events, DISE; 12-Item General Health Questionnaire, GHQ-12; Gratitude Questionnaire, GQ-6; grade point average, GPA; Five Facet Mindfulness Questionnaire, FFMQ; Generalized Anxiety Disorder Scale; GAD; General Self-Efficacy Scale, GSES; General Health Questionnaire, GHQ; heart rate, HR; Liebowitz Social Anxiety Scale, LSAS; negative emotion list, NEI; Mindfulness Attention Awareness Scale, MAAS; Maslach Burnout Inventory, MBI; Mini-International Neuropsychiatric Interview, MINI; Multidimensional Scale of Perceived Social Support, MSPSS; Medical Outcome Study Sleep Scale, MOS SLP9; Patient Health Questionnaire for Depression and Anxiety, PHQ-4; Rumination and Reflection Questionnaire, RRQ; Perceived Medical School Stress Scale, PMSS; Perceived Stress Questionnaire, PSQ; Pittsburgh Sleep Quality Index, PSQI; Positive and Negative Affect Schedule, PANAS; Perceived Stress Scale, PSS; Patient-Reported Outcome Measurement Information System, PROMIS; Psychosocial Well-Being Index-Short Form, PWI-SF; Penn State Worry Questionnaire, PSWQ; Resilience Scale, RS; Satisfaction with Body Parts Scale, SBPS; Social Connectedness Scale, SCS; Social Connectedness Scale-Revised, SCS-R; Self-Compassion Scale, SCS; Self-Rating Anxiety Scale, SAS; Self-Rating Depression Scale, SDS; Sense of Coherence Scale, SOC; Single-Item Measure of Burnout, SIMB; Single-Item Self-Esteem Scale, SISE; systolic blood pressure, SBP; State-Trait Anxiety Inventory, STAI; subjective health complaints, SHC; Subjective Well-Being Scale, SWB; Trait Meta-Mood Scale, TMMS; Satisfaction with Life Scale, SWLS; World Health Organization Quality of Life-Brief Version, WHOQOL-BREF; Warwick–Edinburgh Mental Well-Being scale, WEMWBS.

**Table 3:** Methodological quality of the included studies.

Authors/year/country	Randomization	Allocation concealment	Blinding	Incomplete outcome data	Selective outcome reporting	Other sources of bias
Bai et al. 2020 [24] USA	Random number table	Unclear	Unclear	Yes	Yes	Unclear
Baumgartner and Schneider 2023 [25] USA	Computer-generated random number table	Mention	Subjects	Yes	Yes	Unclear
Cavanagh et al. 2013 [33] USA	Research randomizer website	Computer generated blocked random allocation	Yes (research assistant, participants)	Yes	Yes	Unclear
Chen et al. 2013 [34] USA	Computer-generated random number table	Mention	Yes (research assistant, participants)	Yes	Yes	Unclear
Danitz and Orsillo 2014 [30] USA	Computer-generated random number table	Unclear	Unclear	Yes	Yes	Unclear
Martínez-Rubio et al. 2021 [35] UK	Computer-generated random number table	Mention	Unclear	Yes	Yes	Unclear
Delgado et al. 2010 [36] USA	Computer-generated random number table	Unclear	Unclear	Yes	Yes	Unclear
Dvořáková et al. 2017 [37] USA	Online randomizer	Mention	Yes (research assistant, participants)	Yes	Yes	Unclear
Vibe et al. 2013 [38] NOR	Computer-generated random number table	Unclear	Unclear	Yes	Yes	Unclear
Seppälä et al. 2020 [26] UK	Random number table	Mention	Yes (participants)	Yes	Yes	Unclear
Galante et al. 2020 [32] USA	Computer-generated random number table	Concealed in opaque, sealed envelopes	Yes (student sample)	Yes	Yes	Unclear
Greeson et al. 2014 [31] USA	Research randomizer (www.randomizer.org)	Blocked randomization	Yes (MBSR course instructors)	Yes	Yes	Unclear
Gu et al. 2018 [49] CHA	Computer-generated random number table	Concealed in opaque, sealed envelopes	Yes (participants)	Yes	Yes	Unclear
Hazlett-Stevens and Oren 2016 [40] USA	Mention	Unclear	Unclear	Yes	Yes	Unclear
Huberty et al. 2019 [27] USA	Online randomization tool (randomizer.org)	Mention	Yes (participants)	Yes	Yes	Unclear
Kang et al. 2009 [41] KOR	Mention	Mention	Yes (participants)	Yes	Yes	Unclear
Kvillemo et al. 2016 [42] SWE	Random number table	Mention	Yes (participants)	Yes	Yes	Unclear
Luethcke et al. 2011 [43] USA	Block-randomization procedure	Unclear	No	Yes	Yes	Unclear
Lever Taylor et al. 2014 [44] UK	Block randomization	Mention	Yes (participants)	Yes	Yes	Unclear
Erogul et al. 2014 [45] USA	Random number table	Mention	Yes (participants)	Yes	Yes	Unclear
Simonsson et al. 2013 [29] USA	Random number table	Mention	Unclear	Yes	Yes	Unclear
Ritvo et al. 2021 [28] CAN	1 : 1 block randomization	Concealed in opaque, sealed envelopes	Yes (participants and research assistant)	Yes	Yes	Unclear
Phang et al. 2015 [50] CHA	Random number table	Mention	Yes (participants)	Yes	Yes	Unclear
Recabarren et al. 2019 [46] CH	Computer-generated random number table	Mention	Yes (participants)	Yes	Yes	Unclear
Sun et al. 2021 [47] USA	Random number table	Mention	Yes (participants)	Yes	Yes	Unclear
Song and Lindquist 2015 [48] USA	Computer-generated random number table	Mention	Yes (participants)	Yes	Yes	Unclear
van Dijk et al. 2017 [51] NL	Research randomizer website	Mention	Yes (participants)	Yes	Yes	Unclear
Yang et al. 2018 [53] USA	Research randomizer website	Mention	(participants and research assistant)	Yes	Yes	Unclear
Gu et al. 2017 [39] CHA	Computer-generated random number table	Unclear	Yes (subjects)	Yes	Yes	Unclear
Warnecke et al. 2011 [52] AUS	Random number table	Mention	Yes (participants)	Yes	Yes	Unclear

**Table 4:** Effect sizes of MBSR versus control interventions.

Outcome	No. of studies	No. of sample	Standardized mean difference (95% confidence interval)	Heterogeneity *p* value	*I* ^2^ (%)	Test for overall effect *p* value
Anxious	16 [25, 26, 28–30, 33–36, 38–41, 43–48]	1484	−0.29 (−0.49, −0.09)	*p* ≤ 0.001	72.4	*p* ≤ 0.001
Depression	16 [26, 28, 29, 34–37, 39–44, 47, 48, 51]	1258	−0.32 (−0.62, −0.02)	*p* ≤ 0.001	86.0	*p* ≤ 0.03
Perceived stress	17 [24–28, 30–33, 35, 38, 40, 41, 44, 45, 50–53]	1375	−0.41 (−0.60, −0.29)	*p* ≤ 0.001	70.7	*p* ≤ 0.001
Sleep quality	3 [26–31, 31–37]	280	−0.20 (−0.06, 0.20)	*p* ≥ 0.05	63.4	*p* ≥ 0.05
Mindfulness	15 [25, 26, 28, 30, 31, 33, 35–37, 40, 44, 45, 47, 50, 53]	1180	0.34 (0.08, 0.59)	*p* ≤ 0.001	80.5	*p* ≤ 0.01
Self-kindness	11 [22, 25–27, 30, 35–37, 43, 44, 50]	706	0.57 (0.30, 1.12)	*p* ≤ 0.001	93.2	*p* ≤ 0.04
Social function	5 [26, 33, 37, 40, 48]	453	−0.71 (−2.40, 0.97)	*p* ≤ 0.001	98.1	*p* ≥ 0.05
Subjective well-being	11 [24–26, 31, 32, 36–38, 40, 44, 50]	1050	0.07 (−0.18, 0.32)	*p* ≤ 0.001	78.5	*p* ≥ 0.05
Physical health	6 [26, 33, 35, 39, 40, 43]	504	−0.59 (−1.14, −0.14)	*p* ≤ 0.001	86	*p* ≤ 0.001

**Table 5:** Effect sizes of meta-regression analysis.

	Constant	Coefficient	SE	*t* value	*p* value	95% confidence interval for coefficient	
						UL	LL
Depression							
Recruitment	0.17	−0.21	0.19	1.17	0.32	−0.54	0.21
MBSR duration	−0.09	−0.11	0.22	−0.42	0.50	−0.32	0.26
Control group	0.14	−0.05	0.12	−0.17	0.01	−0.80	0.76
Anxiety							
Recruitment	−0.05	0.07	0.14	0.051	0.80	−0.11	0.12
MBSR duration	0.19	−0.09	0.18	−1.29	0.04	−0.15	0.05
Control group	0.11	−0.12	0.13	−0.36	0.71	−0.29	0.22
Perceived stress							
Recruitment	0.55	−0.20	0.17	−1.33	0.34	−0.50	0.25
MBSR duration	−0.27	0.07	0.60	0.17	0.02	−1.28	1.36
Control group	−0.54	0.19	0.45	2.11	0.50	0.43	1.41
Mindfulness							
Recruitment	0.72	0.25	0.29	0.81	0.46	−0.30	0.60
MBSR duration	0.09	−0.11	0.73	−0.59	0.001	−0.30	0.50
Control group	0.30	−0.06	0.22	−0.12	0.90	−0.47	0.48

**Table 6:** GRADE quality of evidence assessment for the effect of mindfulness-based stress reduction on the symptom management of postoperative side effects among university students.

Quality assessment							Summary of finding table			Quality	Importance
							No of university students		Effect		
Outcome/no of studies	Design	Risk of bias	Inconsistency	Indirectness	Imprecision	Other considerations	MBSR	Control	Relative (95% CI) Absolute		
Anxiety/17	Randomized trials	No serious risk of bias^1^	No serious inconsistency	No serious indirectness	Serious^3^	None	733	705	−0.31 (−0.43, −0.22)	MODERATE	CRITICAL
Depression/17	Randomized trials	No serious risk of bias	No serious inconsistency	No serious indirectness	Serious^3^	None	678	687	−0.19 (−0.98, −0.15)	MODERATE	CRITICAL
Perceived stress/17	Randomized trials	No serious risk of bias^1^	No serious inconsistency	No serious indirectness	Serious^3^	None	333	300	−0.48 (−0.71, −0.26)	MODERATE	CRITICAL
Sleep quality/3	Randomized trials	Serious^1^	No serious inconsistency	No serious indirectness	Serious^3^	Reporting bias^4^	335	333	−0.20 (−0.06, 0.20)	LOW	IMPORTANT
Mindfulness/17	Randomized trials	No serious risk of bias^1^	No serious inconsistency	No serious indirectness	Serious^3^	None	705	646	0.52 (0.11, 0.68)	MODERATE	CRITICAL
Self-kindness/9	Randomized trials	No serious risk of bias	No serious inconsistency	No serious indirectness	Serious^3^	None	425	410	0.41 (−0.57, 0.95)	MODERATE	CRITICAL
Social function/5	Randomized trials	No serious risk of bias	No serious inconsistency	No serious indirectness	Serious^3^	Reporting bias^4^	57	66	−0.71 (−2.40, 0.97)	MODERATE	IMPORTANT
Subjective well-being/11	Randomized trials	No serious risk of bias^1^	No serious inconsistency	No serious indirectness	Serious^3^	None	574	600	0.15 (−0.45, 0.74)	MODERATE	IMPORTANT
Physical health/10	Randomized trials	Serious^1^	No serious inconsistency^2^	No serious indirectness	Serious^3^	Reporting bias^4^	272	262	−0.59 (−1.14, −0.14)	LOW	IMPORTANT

^1^Some RCTs did not mention using the blind method or randomized grouping. ^2^Similarity of point estimates, extent of overlap of confidence intervals, and statistical criteria are poor. ^3^Confidence in estimates of effect is poor, small sample size, and SRCTs do not calculate the optimal information size. ^4^Small sample and asymmetry of the funnel plot.
